# Supplementary material for: Regulation of Mitochondrial Metabolism by Mfn1 Gene Encoding Mitofusin Affects Cellular Proliferation and Histone Modification
Source: Cells. 2025 Jul 2;14(13):1015. doi: 10.3390/cells14131015 (PMC12248831; doi:10.3390/cells14131015)
Supplement: Supplementary file 1 [file cells-14-01015-s001.zip › Supplementary Figures.pdf]

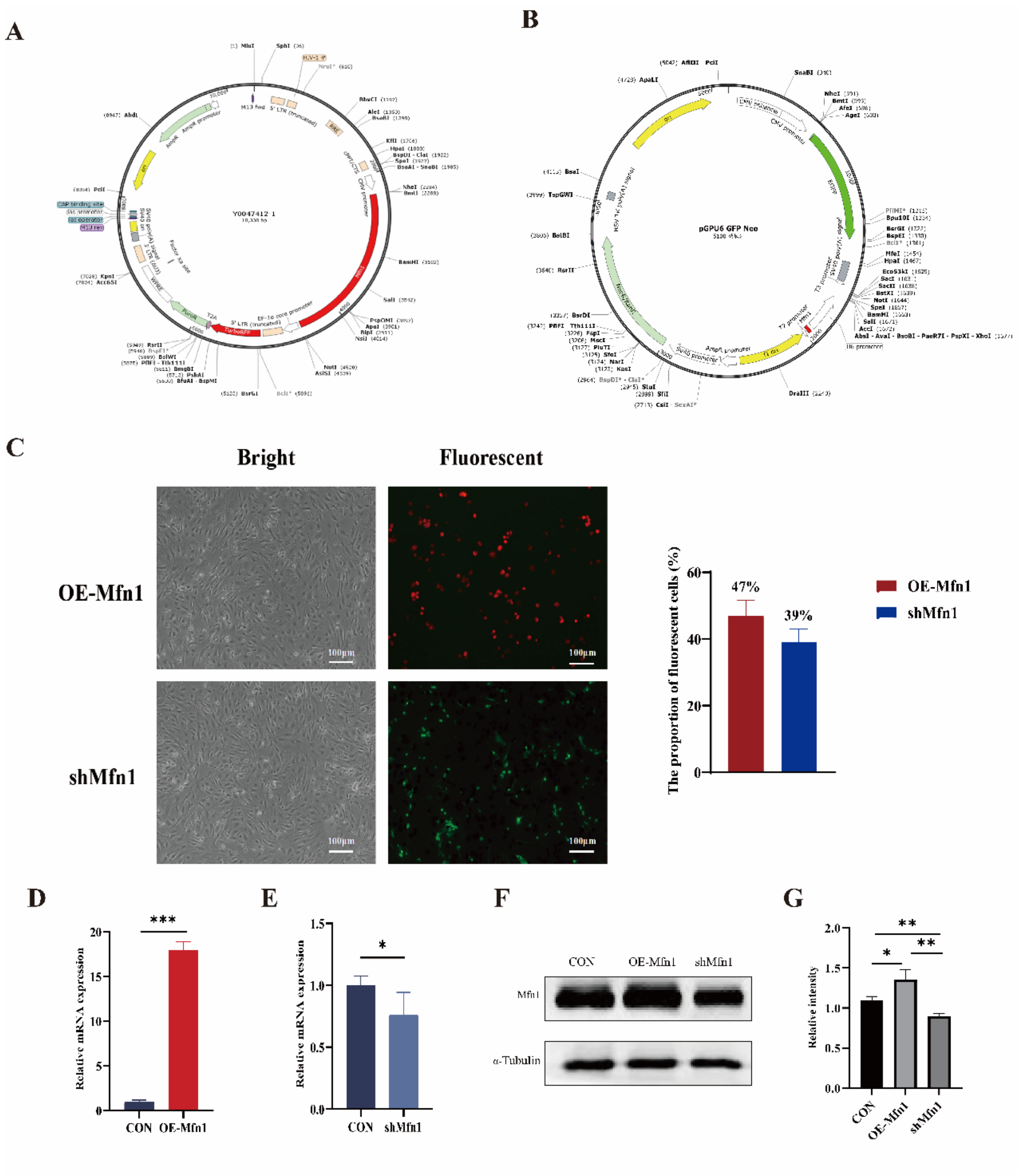

Figure S1. OE-Mfn1 and shMfn1 vector diagrams and transfection efficiency detection. (**A**) OE-Mfn1 vector diagram. (**B**) shMfn1 vector diagram. (**C**) Transfection of bovine fetal fibroblasts with OE-Mfn1 and shMfn1 vectors and statistical analysis of transfection efficiency. (**D**) RT-qPCR detection of Mfn1 gene expression in bovine fetal fibroblasts after transfection with the OE-Mfn1 vector. (**E**) RT-qPCR detection of Mfn1 gene expression in bovine fetal fibroblasts after transfection with the shMfn1 vector. (**F**) Western blot detection of MFN1 protein expression in CON, OE-Mfn1 and shMfn1 groups, respectively. (**G**) Grayscale analysis of MFN1 protein in CON, OE-Mfn1 and shMfn1 groups, respectively. *P<0.05, **P<0.01, ***P<0.001.

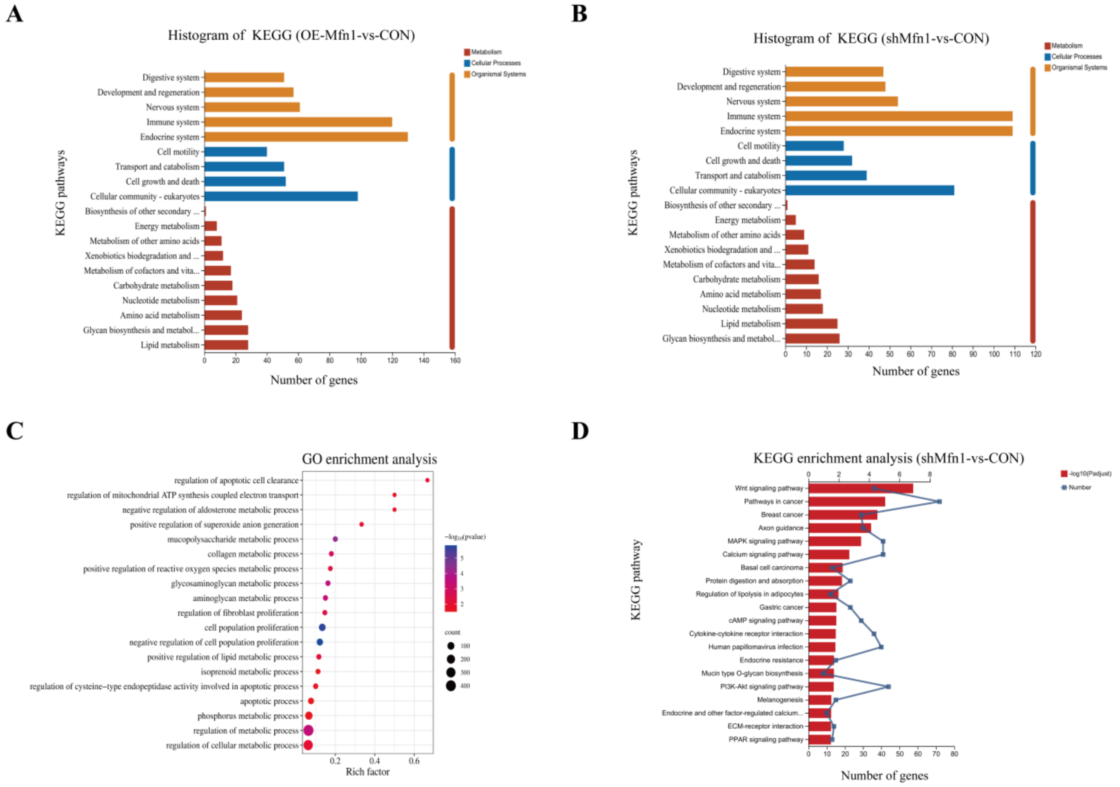

Figure S2. RNA-seq analysis of OE-Mfn1 and shMfn1 cells.

(**A**) KEGG functional annotation analysis of differentially expressed genes between CON and OE-Mfn1 groups. (**B**) KEGG functional annotation analysis of differentially expressed genes between CON and shMfn1 groups. (**C**) GO functional enrichment analysis of differentially expressed genes between CON and shMfn1 groups. (**D**) KEGG pathway enrichment analysis of differentially expressed genes between CON and shMfn1 groups.

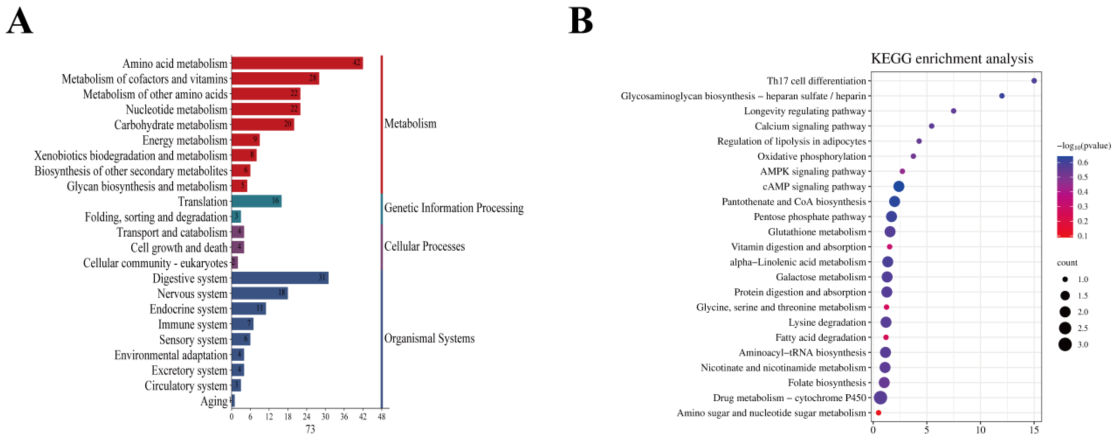

Figure S3. Metabolomic analysis of shMfn1 group cells.

(**A**) KEGG functional enrichment analysis of differentially expressed metabolites between CON and shMfn1 groups. (**B**) KEGG pathway enrichment analysis of differential metabolites between CON and shMfn1 groups.
