# Supplementary material for: Regulation of Mitochondrial Metabolism by Mfn1 Gene Encoding Mitofusin Affects Cellular Proliferation and Histone Modification
Source: Cells. 2025 Jul 2;14(13):1015. doi: 10.3390/cells14131015 (PMC12248831; doi:10.3390/cells14131015)
Supplement: Supplementary file 1 [file cells-14-01015-s001.zip › Supplementary Table.pdf]

Supplementary Table S1. List of RT-qPCR specific primers used this study.

Gene name	Forward primer (5'-3')	Reverse primer (5'-3')
Mfn1	GCTGCATACAGACAGACAGCCT	GGTAATGCCCTGTCTCAGGGCT
Pcna	AGTCACATTGGAGATGCTGTTGTA	AGCTGAACTGGTTCATTCATCTCTA
Cdk1	TGTTCAGCAGAGTCAGGGATGG	GAGGACAGCTGTGTAAAGCTTGAGG
Cdk2	TGGGTCCATCAAGCTAGCAGA	TGGAGTAGTATTTGCAGCCCAGA
Mk167	CCAGGCTGCTGTAAATGCTTC	CGGACACCCTGTCATTGTTC
Cdkn1a	TTGCACTTTGAATAACAGCTGAAGG	CAAGTGGTTCAGCGCAAGGA
Cdkn2a	CGTGCTGATGGCTAGTGAGGA	CCATCATCATCACCTGGTCTAGGA
Bad	GAGCAGAGTGAGCAGGAA	CCTCCGTCTCTTCATTATCC
Casp3	CCAGGGTGCCCAGGACTTTA	GTGTCCCATCTCTGGCCTTCA
Casp9	CCCAGGGACTTCTGGTGGTTAG	TCTGCACACAAGGCTCTGCTC
Cycs	GGTTGCACCAACACCGGTA	CACTGGGCACACTTCTGAACA
Bcl2	CTGTGGATGACCGAGTACCTGAAC	AGACTGAGCAGTGCCTTCAGAGAC
Xiap	GGGAACAACATGCTAAATGG	CTGCAACCAGAACCTCAAGT
Kdm1a	GCTACATCTTACCTTAGTCATC	AGTTCCTTCAGTTCCTCCT
Kdm1b	GAAGGTGGAAGAATAACTGAC	TGCTGAGATGGAACTGAAG
Kdm6a	ACAAAACTGGCAACATAATACAG	AAAGTTGACCAAATAAAGACTTA
Kdm6b	CTCTTTTCTTTTTAAGCGTGAAACAG	CAAAAAACAACCGACAAAACGA
Kat2a	TGTCGGAGGATGAGATTAAC	TGAAGATGGAGCGGAGAA
Kat2b	AACACGACATCCTGAACTT	ATCTGCCGAACTCCATCT
Kat7	CTGACGAGCGAGTATGAC	TTCTTGCCATCCACTTCAA
Sirt1	AGGTTAGGTGGTGAATATGC	AAGAATCTGGTGGTGAAGTT
Sirt2	AGGCTCAGGACTCAGATT	ATGAAGTAGTGGCAGATGG
Sirt3	CAGCATCCTCCAGCAGTA	CCTTCTCGTGTAGCAATCG
Sirt4	TACTCAGGTTACAGGTTCATC	TTAATGGCAGCAACTCTCC
Sirt5	GTGTGAAGAGGCAGGATG	GGATAGACCACGGAGGAA

Sirt6	GAGGAGTTGGAGCAGAAG	GCATCTTACACTTGACACAT
Sirt7	CGAGAGGAGCAACAGAGA	TCAGGTCAGCAGCACTAA
Gapdh	GATGGTGAAGGTCGGAGTGAAC	GTCATTGATGGCGACGATGT
